# Measuring partnership synergy and functioning: Multi-stakeholder collaboration in primary health care

**DOI:** 10.1371/journal.pone.0252299

**Published:** 2021-05-28

**Authors:** Ekaterina Loban, Cathie Scott, Virginia Lewis, Jeannie Haggerty

**Affiliations:** 1 St. Mary’s Research Centre, Montreal, Quebec, Canada; 2 Department of Family Medicine, McGill University, Montreal, Quebec, Canada; 3 Department of Community Health Sciences, University of Calgary, Alberta, Canada; 4 Australian Institute for Primary Care & Ageing, La Trobe University, Melbourne, Australia; Queensland University of Technology, AUSTRALIA

## Abstract

In primary health care, multi-stakeholder partnerships between clinicians, policy makers, academic representatives and other stakeholders to improve service delivery are becoming more common. Literature on processes and approaches that enhance partnership effectiveness is growing. However, evidence on the performance of the measures of partnership functioning and the achievement of desired outcomes is still limited, due to the field’s definitional ambiguity and the challenges inherent in measuring complex and evolving collaborative processes. Reliable measures are needed for external or self-assessment of partnership functioning, as intermediate steps in the achievement of desired outcomes. We adapted the Partnership Self-Assessment Tool (PSAT) and distributed it to multiple stakeholders within five partnerships in Canada and Australia. The instrument contained a number of partnership functioning sub-scales. New sub-scales were developed for the domains of communication and external environment. Partnership synergy was assessed using modified Partnership Synergy Processes and Partnership Synergy Outcomes sub-scales, and a combined Partnership Synergy scale. Ranking by partnership scores was compared with independent ranks based on a qualitative evaluation of the partnerships’ development. 55 (90%) questionnaires were returned. Our results indicate that the instrument was capable of discriminating between different levels of dimensions of partnership functioning and partnership synergy even in a limited sample. The sub-scales were sufficiently reliable to have the capacity to discriminate between individuals, and between partnerships. There was negligible difference in the correlations between different partnership functioning dimensions and Partnership Synergy sub-scales. The Communication and External Environment sub-scales did not perform well metrically. The adapted partnership assessment tool is suitable for assessing the achievement of partnership synergy and specific indicators of partnership functioning. Further development of Communication and External Environment sub-scales is warranted. The instrument could be applied to assess internal partnership performance on key indicators across settings, in order to determine if the collaborative process is working well.

## Introduction

Multi-stakeholder partnerships have been defined as a “voluntary and collaborative relationship … between various [stakeholders from different organizations] … in which all participants agree to work together to achieve a common purpose or undertake a specific task and to share risks, responsibilities, resources, competencies and benefits” [1]. Partnerships can help people and organizations generate outcomes that are greater than those that can be achieved working independently [2–4]. Partnerships continue to be widely embraced with governments and funding bodies mandating partnerships as an essential element of the programs and initiatives that they support [3,5,6].

In practice, however, partnerships can generate a great deal of frustration, as different organizations and individuals frequently struggle to achieve measurable and mutually beneficial outcomes [3,5,7–9]. These problems are not surprising given that partnerships are resource-intensive, take time and require governance, procedures and processes that are very different from how independent organizations are run [3]. Measures of partnership functioning and partnership self-assessment tools can help organizations assess how the partnership is evolving, benchmark against external criteria, stay accountable to the partnership’s stakeholders and funders, and monitor and maximize the effectiveness of the collaborative effort [6,10–12].”

This article presents findings from a survey of five multi-stakeholder partnerships involving decision makers, academic representatives, clinicians and organizational representatives with an interest in improving primary health care (PHC) accessibility. This quantitative study was part of a larger mixed methods sequential exploratory study to understand the processes that enhance multi-stakeholder partnerships in PHC service delivery. The qualitative phase entailed a comparative qualitative case study exploring, in a smaller sample of partnerships, the different manifestations of partnership synergy, different types of partnership resources and broad categories of partnership processes relevant to multi-stakeholder partnerships in PHC. Based on qualitative findings, approximately three-to-four years into the partnerships, we adapted an existing quantitative tool to measure different functions of partnerships and specifically to measure the concept of partnership synergy as an intermediate measure of partnership effectiveness. This article reports on the metric performance of the adapted measures of partnership functioning and partnership synergy.

### Partnership evaluation and synergy

Some scholars and practitioners have argued that various dimensions of partnership functioning—including partner relationships, governance, leadership, management, use of resources, and the external environment–influence the achievement of the planned partnership outcomes [3,13,14]. The literature offers different definitions and conceptualizations of partnership effectiveness, and complex processes and dynamics are inherently difficult to evaluate [9,15–18].

The notion of “partnership synergy” has recently been proposed as an intermediate outcome between partnership functioning and the achievement of the planned ultimate outcomes [3]. Synergy is viewed as the key mechanism on the pathway by which partnerships gain advantage over the independent work of individual stakeholders working towards the same goals [3]. Partnerships are said to be synergistic when they leverage resources successfully and mobilize the complementary knowledge and expertise of all partners, with the effect that the whole becomes greater than the sum of the parts [2,3]. The assumption is that if a partnership achieves a high level of synergy, it is better placed to achieve desired partnership goals. The advantage of measuring synergy is that it is manifested (or not) earlier and is more directly attributed to partnership functioning than the ultimate outcomes [3].

Two validated scales of partnership synergy have been developed. Both scales purport to assess the degree to which the partnership has maximized its synergy but each measures partnership synergy differently. The Weiss *et al*. scale [19] conceptualizes synergy as a product of good quality partnership processes [3], whereas the Jones synergy scale measures synergy as both a partnership process and a partnership product [20]. Both synergy scales are embedded in instruments that include other measures of different partnership functioning dimensions [7,19,21].

### Partnership Self-Assessment Tool (PSAT)

We selected as the study instrument the Partnership Self-Assessment Tool (PSAT) [22] which contains the Weiss *et al*. partnership synergy scale. The PSAT also measures the various dimensions of partnership functioning including leadership, efficiency, administration and management, non-financial resources, financial and other capital resources, decision-making, benefits and drawbacks of participation and overall satisfaction with participation [22]. The PSAT was developed by public health scholars for use by groups working to promote health and well-being in their communities [19,23,24]. Although very similar to another partnership instrument that integrated the Weiss *et al*. and the Jones partnership synergy scales (Partner Questionnaire) [20], we primarily used the PSAT, but integrated some of the nuances from the Partner Questionnaire. The PSAT is in the public domain and no conditions are specified for its use [25]. The PSAT was “provided to partnerships at no charge by the Center for the Advancement of Collaborative Strategies in Health at The New York Academy of Medicine with funding from the W.K. Kellogg Foundation” [23].

We made some adaptations to the PSAT. First, we modified the Partnership Synergy scale, incorporating items from the Weiss *et al*. and the Jones partnership synergy scales [20,22]. Second, we developed new measures of communication and external environment and we adapted the content of other sub-scales based on the findings of the comparative case study of two of the partnerships. The objective of this study was to assess the reliability of the adapted PSAT instrument and sub-scales, specifically the measures of partnership synergy, to assess its capacity to discriminate between partnerships, and to provide conclusions that were coherent with the independent qualitative developmental evaluation that ran in parallel to the mixed methods study.

## Methods

We used a cross-sectional survey design, distributing a self-administered questionnaire to multiple stakeholders within five collaborative local partnerships in three Canadian provinces (Quebec, Ontario and Alberta) and two Australian states (Victoria and New South Wales). The survey took place between September 2018 and March 2019. Ethics approval for the study was obtained from the St. Mary’s Hospital Center Research Ethics Committee (No. SMHC-13-30C). All participants provided consent prior to data collection.

### Study population and sampling

The five partnerships were established in 2014 within a Canada-Australia research program entitled “Innovative Models Promoting Access-to-Care Transformation” (IMPACT). The program aimed at implementing organizational interventions to improve access to appropriate PHC for vulnerable populations [26]. Using a participatory action research approach, each partnership identified local PHC access need, and selected, adapted, implemented and evaluated an organizational intervention to address the need. The ultimate outcome was to meet the health care needs of the specific population through PHC and avoid unnecessary emergency room use and hospitalizations.

Each partnership involved a combination of the following stakeholders: policy makers, clinicians and other health and community service providers, health system administrators, university researchers and research coordinators, and, in some cases, members and representatives of vulnerable populations [26]. The premise of the program was that a strong partnership was key to mobilizing the resources for the pilot intervention and ultimately sustaining it, if proved potentially effective. Each IMPACT local partnership was unique, based upon the specific context within which it unfolded, the local access need that the partnership tried to address, tailored processes and requirements that were required to meet this need, and the relationships that formed to move the work forward. As a result, the interventions in each partnership were different both in focus and in the ways in which the partnerships evolved. See Table 1 for an overview of interventions in five partnerships.

**Table 1:** Overview of interventions in five partnerships [adapted from 26,27].

Partnership Title	Intervention objective	Target population	Intervention type
**Service Linkage**	To identify vulnerable individuals who are likely to benefit from better access to PHC* and to successfully link them with PHC practices.	Vulnerable individuals who are clients of community-based chronic disease services. Clients had at least one the of the following characteristics: low socioeconomic status, social isolation due to geographic distance/public transport, chronic illness of developmental disability.	Community-based chronic disease services identify patients without a primary care provider. A broker then links identified patients to one of a panel of volunteer family practitioners.
**Community Health Resources**	To increase primary care providers’ awareness of and referral to community resources for socially vulnerable patients, and to assist these patients in overcoming barriers to reach the services.	Chronically ill primary care patients not receiving available community services that would optimize disease management.	A lay patient navigator within primary care practices to facilitate access to community-based health and social resources for vulnerable patients–those experiencing social barriers.
**Diabetes Self-management**	To identify the enabling factors and assess the impact of web-based information and education tools that support chronic disease self-management in a vulnerable PHC population attending practices in low socioeconomic areas.	Patients with poorly controlled diabetes attending practices in low socioeconomic neighborhoods. Targeted subgroups: low socioeconomic status, culturally and linguistically diverse communities, refugee and humanitarian entrants.	A website that provides information and referral options to support diabetes self-management, facilitated by practice nurses at a health check visit in the PHC practice.
**Primary Care Connection**	To enhance the retention of vulnerable patients who were recently assigned to a family physician from a centralized wait list.	Unattached patients in two high deprivation neighborhoods (e.g., low income, high unemployment, low social support), especially those with mental health diagnoses or poor health.	A telephone outreach service by lay volunteers to help prepare for their first visit and explain important access-related issues, in order to facilitate forging an on-going relationship with their care provider.
**Community Outreach**	To identify the components of outreach and colocation of PHC services, as identified by vulnerable populations, that contribute to making these services more approachable and engaging.	Individuals and groups of vulnerable populations living in a geographic area with few PHC services but high concentration of marginalized populations, including: recent immigrants, Aboriginal people, seniors, the homeless.	Mobile (pop-up) PHC services, held in different locations, for vulnerable patients in an underserved urban area.

* PHC—Primary Health Care.

The partnerships met the following criteria for the PSAT use: the partnerships had been in existence for at least six months, had at least five active partners, had continually worked together to develop and modify strategies in order to achieve their goals, and had begun to implement their plans [23]. We used a census sample of all members of the five local partnerships at the time of data collection. All 61 stakeholders who were active in the partnerships at the time of administration were considered eligible and were invited to participate.

### Instrument adaptation and administration

We adapted the validated Partnership Self-Assessment Tool (PSAT) [22,24] as outlined in supplementary material S1 Appendix. First, we made small linguistic adaptations to fit the PHC study context, by reflecting the roles and organizational expertise of the stakeholders in PHC partnerships. Second, we adapted the PSAT based on analysis of the case studies in two partnerships with the objective of understanding and describing how partnership processes and approaches to enhance partnerships were perceived and experienced by local partnership stakeholders. We selected PSAT sub-scales and items that corresponded to qualitative codes from the case study and excluded items that were deemed not relevant. In addition, we supplemented synergy items from the PSAT with synergy items from the Partnership Questionnaire [20] to capture more information on partnership processes. The adapted instrument retained the following dimensions of partnership functioning from the PSAT: Decision-making (four items), Leadership (11 items), Administration & Management (11 items), Non-financial Resources (six items); Financial & Other Resources (three items); and Resource Utilization (three items, referred to as “Efficiency” in the PSAT).

We added elements that emerged in qualitative data but were not part of the PSAT: new proposed sub-scales for Communication (three items) and External Environment (two items). The case studies spoke to the importance of communication as an integral dimension of partnership functioning and the critical role of external influences.

Partnership Synergy was assessed using two sub-scales: 1) the adapted Partnership Synergy Processes sub-scale incorporating five items from the eight-item synergy scale developed by Jones and Barry [20]; and 2) the adapted Partnership Synergy Outcomes sub-scale retaining two items from the nine-item synergy scale by Weiss *et al* [19]. The items from these original scales were selected based upon the relevance to the types of partnerships highlighted throughout the qualitative inquiry, and with a view to reduce participant burden. More items were retained from the Jones than the Weiss *et al*. synergy scale as they were deemed more relevant in light of our overall aim to understand the processes and approaches of successful partnerships. In addition, they were more appropriate in our sample of partnerships that were underway and that exhibited varied levels of engagement of community-based stakeholders representing the target population. The partnerships were at a stage in their development when it was premature or impossible to evaluate elements of the Weiss *et al*. scale, such as the ability “to respond to the needs and problems of the community” or “to implement strategies that are most likely to work in the community” [22]. Respondents were also asked to assess the extent to which the goal of developing a meaningful partnership had been achieved (1 item).

The majority of questions offered five-point Likert response options. We changed the order of response options to present the most negative response first, in order to reduce the commonly observed positive bias in responding [28]. The use of the “don’t know” response option was minimized, to reduce the occurrence of missing data and on the grounds that there was little reason to suppose that respondents would not know. The instrument captured the stakeholders’ assessments of the partnerships: some questions elicited the individual stakeholders’ own perceptions; others, the perspectives of the stakeholders’ respective organizations. The respondents were asked to provide an assessment of the partnership at the point in time when the tool was administered.

Finally, the questionnaire included questions about perceived Benefits (11 items) and Drawbacks (5 items) of participation to their respective organizations, and an overall assessment of benefits compared to drawbacks (1 item). It also elicited descriptive information about the stakeholder (type, of role in the organization, length of time in the partnerships, frequency of engagement).

The questionnaire was tested through cognitive interviewing [29] with three professionals familiar with PHC terminology, to verify that the content of the questions was understood as intended, to select the most appropriate response scales, and to ascertain whether the language, the length and format were appropriate. Modifications were mostly confined to amending response scales and choosing the appropriate terminology in French. The translation into French was performed by the first author (EL) and verified by a member of the research team familiar with the terminology and the IMPACT program context.

The questionnaire was self-administered according to existing guidelines in the survey literature [28]. Both paper and web-based questionnaires were offered, and in either English or French. The electronic questionnaire was hosted on the *Qualtrics* [30] online survey platform.

### Data analysis

The unit of analysis for analysing the metric properties of the instrument was the individual respondent (n = 54), whereas the unit of analysis for partnership functioning and synergy was the aggregate of individual responses within a given partnership (n = 5). We preferred the median and IQR as the most appropriate way of representing central tendency and spread given the categorical nature of the data.

We used descriptive statistics to characterize the sample and the distribution of the items and sub-scale scores. The scores for each sub-scale were derived through unweighted mean and medians of all the component items; scores for a respondent were calculated only if at least 50% of the items in the scale had been completed. Another method for handling the missing data was pairwise deletion. The small sample size precluded classical factor analysis. Instead, the analysis focused on examining some of the metric properties that may compromise precision and reliability. We looked for floor and ceiling effects, using a threshold ≥ 45% respondents in the lowest or highest response option. These items were tagged for exclusion as they indicated reduced capacity to discriminate between respondents. We used Spearman correlation coefficients to estimate the correlation between items within purported sub-scales, and between sub-scales, specifically dimensions of partnership functioning and partnership synergy. We examined internal consistency of the purported sub-scales by calculating the Cronbach’s Alpha.

At the partnership level, the score for each scale was calculated by aggregating the scale scores of the component member respondents within each partnership. For partnership synergy, the two sub-scales of Partnership Synergy Processes and Partnership Synergy Outcomes were analysed separately, at the level of individual respondents. However, given that they were highly correlated with each other and demonstrated similar correlations with dimensions of partnership functioning, both sub-scales were combined at the level of the partnerships to provide a single score of Partnership Synergy. We used the non-parametric Kruskal–Wallis test to determine if we were able to detect statistically significant differences in sub-scale scores between any of the partnerships. The ranks of the scores were also derived. SPSS 23 for Windows [31] was used for data analyses.

In lieu of statistical testing of predictive and convergent validity, we used consensus ranking from two external content reviewers who had conducted the independent longitudinal developmental evaluation of the five IMPACT partnerships as part of the IMPACT program’s effort to support the partnerships. They ranked the five partnerships based on the operational definitions and item content of the Partnership Synergy sub-scales and of the following partnership functioning sub-scales: Communication, Decision-making, Problem-solving, Resource Utilization and External Environment. The qualitative and quantitative ranks were compared. Finally, we correlated the score of Partnership Synergy with the partnership aggregate assessment of the extent to which developing a meaningful partnership had been achieved.

## Results

### Sample characteristics

The response rate was 90% (55/61), of which 62% (34) were online and 38% (21) were in paper format; 82% (45) in English and 18% (10) in French. One respondent was subsequently excluded from analysis due to comments on the questionnaire admitting that they picked answers at random. Our final sample was therefore 54 participants (see supplementary material S1 Dataset for dataset). Of note, two partnerships, namely Primary Care Connection and Community Health Resources, participated in both phases of this mixed methods study, with 15 partnership stakeholders participating in both the qualitative phase (involving interviews) and this quantitative phase (entailing a survey).

The partnerships varied in their size and composition. Table 2 outlines the descriptive characteristics of each of the partnerships. Academic representatives constituted the largest single group of stakeholders in each partnership. Of the five partnerships, the Community Health Resources partnership had the largest representation of community stakeholders (representing community organizations and patients).

**Table 2:** Study sample characteristics per partnership (*N* = 5).

Partnerships	Total number of participants (*N = 54*)	Gender	Mean length of time in partnership	Min/max length of time in partnership	Main role
		Percent female			
**Service Linkage**	9	78% (7)	3,8 years	2–6 years	Academic representative– 4
					Decision-maker– 1
					Health care manager– 4
**Community Health Resources**	19	68% (13)	2,6 years	1–4 years	Academic representative– 5
					Community organization representative– 3
					Decision-maker– 2
					Health care manager– 4
					Patient representative– 3
					Primary care physician– 2
**Diabetes Self-management**	7	71% (5)	3,1 years	0.7–5 years	Academic representative– 3
					Community representative– 1
					Decision-maker– 1
					Health care manager– 1
					Primary care physician– 1
**Primary Care Connection**	11	91% (10)	3 years	1–5 years	Academic representative– 5
					Decision-maker– 1
					Health care manager– 3
					Patient representative– 1
					Primary care physician– 1
**Community Outreach**	8	86% (7)	2,8 years	2,5–5 years	Academic representative– 6
					Decision-maker– 1
					Health care manager– 1

Five participants did not finish completing the questionnaire for a variety of reasons (stopping at questions 14 (health care manager), 14 (academic representative), 15 (academic representative), 21 (health care manager) and 23 (patient representative) of a total of 76 questions). The missing data rates per question were calculated only on the sample of respondents who completed the questionnaire to that point. Missing data varied from 0% to 18% and the median was 2%, which was under the conventional missing rate of 10% [32]. The missing data rates higher than 10% occurred in the sub-scales of Financial Resources, Resource Utilization and External Environment, suggesting these may be problematic metrically. Ceiling effects were observed in two of the 65 Likert-scale questions in the sub-scales of Decision-making and Leadership. No floor effects were present.

### Partnership functioning and synergy

Table 3 provides descriptive statistics for the dimensions of partnership functioning. We observed that the responses on all dimensions were skewed towards positive evaluations.

**Table 3:** Score distribution for the partnership functioning and partnership synergy sub-scales (*N* = 54). Responses to various five-point Likert scales response options have a negative (1) to positive (5) valence.

#	Sub-scale	Means (SD)	Medians (IQD)	Range of scores
**Partnership Functioning**				
**1.**	**Communication (2 items)**	3.60 (0.60)	3.50 (1)	2.50–4.50
**2.**	**Decision-making (4 items)**	3.75 (0.71)	4.00 (0.94)	2.00–4.75
**3.**	**Leadership (11 items)**	3.83 (0.84)	3.86 (1.39)	1.91–5.00
**4.**	**Administration & Management (11 items)**	3.77 (0.71)	3.82 (1.09)	1.83–5.00
**5.**	**Non-financial Resources (6 items)**	3.73 (0.51)	3.67 (0.50)	2.17–4.83
**6.**	**Financial Resources (3 items)**	4.07 (0.66)	4.00 (0.50)	2.50–5.00
**7.**	**Resource Utilization (3 items)**	4.07 (0.90)	4.33 (1.00)	1.67–5.67
**8.**	**External Environment (2 items)**	3.56 (0.67)	3.50 (1.00)	1.00–5.00
**Partnership Synergy**				
**1.**	**Partnership Synergy Outcomes (2 items)**	3.52 (0.74)	3.50 (1)	2.00–5.00
**2.**	**Partnership Synergy Processes (5 items)**	3.71 (0.65)	3.60 (0.80)	2.00–4.80

Table 4 presents summary information on the Spearman rank correlations between the items within various dimensions of partnership functioning and Partnership Synergy (see supplementary material S2 Appendix for table of individual correlations). We used the following cut-offs for interpreting the scores: .10 –weak correlation; .30 –moderate; .50 –strong [33]. Despite our limited sample size, we examined the correlations to shed light on a number of assumptions that relate to the validity and reliability of measures. We observed that items within the following sub-scales met the assumption of item-convergent validity by being strongly correlated (>0.50) within the sub-scale: Leadership, Administration & Management, Financial Resources and both Partnership Synergy sub-scales. The assumption of item-discriminant validity states that the correlation of items within a hypothesized sub-scale will be higher than with items in other sub-scales. We observed that correlations between items within the following sub-scales were higher than with other sub-scales: Leadership, Administration & Management, Financial Resources and both Partnership Synergy sub-scales.

**Table 4:** Spearman rank correlations between items within each of the sub-scales and between sub-scales of dimensions of partnership functioning and partnership synergy, showing the average, minimum and maximum correlation coefficients, and a measure of internal consistency (*N* = 54).

#	Sub-scale	Average within sub-scale correlation (means)	Average within sub-scale correlation (medians)	Range (min-max)	Cronbach’s α	Average correlation with items in other sub-scales (medians)
**Partnership Functioning**						
**1.**	**Communication (2 items)**	0.36		-	0.49	0.39
**2.**	**Decision-making (4 items)**	0.45	0.44	0.24–0.63	0.77	0.47
**3.**	**Leadership (11 items)**	0.68	0.71	0.34–0.89	0.95	0.53
**4.**	**Administration & Management (11 items)**	0.59	0.59	0.35–0.79	0.93	0.48
**5.**	**Non-financial Resources (6 items)**	0.36	0.40	-0.14–0.64	0.79	0.40
**6.**	**Financial Resources (3 items)**	0.62	0.50	0.50–0.86	0.83	0.18
**7.**	**Resource Utilization (3 items)**	0.39	0.28	0.15–0.74	0.73	0.39
**8.**	**External Environment (2 items)**	-0.28		-	-0.74	-0.06
**Partnership Synergy**						
**1.**	**Partnership Synergy Outcomes (2 items)**	0.54		-	0.69	0.46
**2.**	**Partnership Synergy Processes (5 items)**	0.59	0.65	0.43–0.69	0.89	0.48

Finally, reliability of a scale is assumed if each item has an approximately equivalent correlation within the hypothesized sub-scale and contributes approximately the same proportion of information to the total score. The low and variable correlations in Communication and External Environment translated into a low internal consistency that failed to meet the criterion of ≥0.70 [34]. Cronbach’s α values demonstrated good internal consistency and reliability for most other sub-scales, although the very high within-scale correlation and high internal consistencies in the Leadership, Administration & Management suggest redundancy in the items.

Table 5 displays correlations between the various dimensions of partnership functioning and Partnership Synergy, as assessed using Spearman’s rank correlation coefficient. Together, Tables 4 and 5 demonstrate that the new sub-scales of Communication and External Environment did not perform well metrically, on assumptions of item-convergent validity, item-discriminant validity, and internal consistency. We subsequently excluded these sub-scales from our partnership-level analysis.

**Table 5:** Spearman rank correlations between partnership synergy outcomes, partnership synergy processes, and the different dimensions of partnership functioning (*N* = 54). Values shaded in light grey denote weak correlations, in dark grey–strong correlations.

	Partnership Synergy Outcomes	Partnership Synergy Processes	Communication	Decision-making	Leadership	Admin & Mgmt	Non-financial Resources	Financial Resources	Resource Utilization	External Environment
**Partnership Synergy Outcomes**		0.61	0.34	0.60	0.69	0.60	0.39	0.18	0.46	0.15
**Partnership Synergy Processes**	0.61		0.44	0.59	0.60	0.48	0.51	0.35	0.45	0.26
**Communication**	0.34	0.44		0.47	0.50	0.45	0.38	0.32	0.39	0.10
**Decision-making**	0.60	0.59	0.47		0.65	0.52	0.40	0.07	0.29	0.25
**Leadership**	0.69	0.60	0.50	0.65		0.74	0.42	0.13	0.53	-0.08
**Administration & Management**	0.60	0.48	0.45	0.52	0.74		0.42	0.05	0.50	-0.06
**Non-financial Resources**	0.39	0.51	0.38	0.40	0.42	0.42		0.56	0.32	-0.21
**Financial Resources**	0.18	0.35	0.32	0.07	0.13	0.05	0.56		0.28	-0.18
**Resource Utilization**	0.46	0.45	0.39	0.29	0.53	0.50	0.32	0.28		-0.17
**External Environment**	0.15	0.26	0.10	0.25	-0.08	-0.06	-0.21	-0.18	-0.17	

In addition, we observed that the shortened Partnership Synergy Outcomes and Partnership Synergy Processes sub-scales were strongly correlated with each other (r = 0.61), and there were few differences in how each of them correlated with the different dimensions of partnership functioning. We consequently chose to collapse the two synergy sub-scales together to a single Partnership Synergy scale (Partnership Synergy) for the analysis of associations between Partnership Synergy and functioning dimensions at the partnership level.

### Partnership functioning and synergy at the partnership level

**Fig 1:**
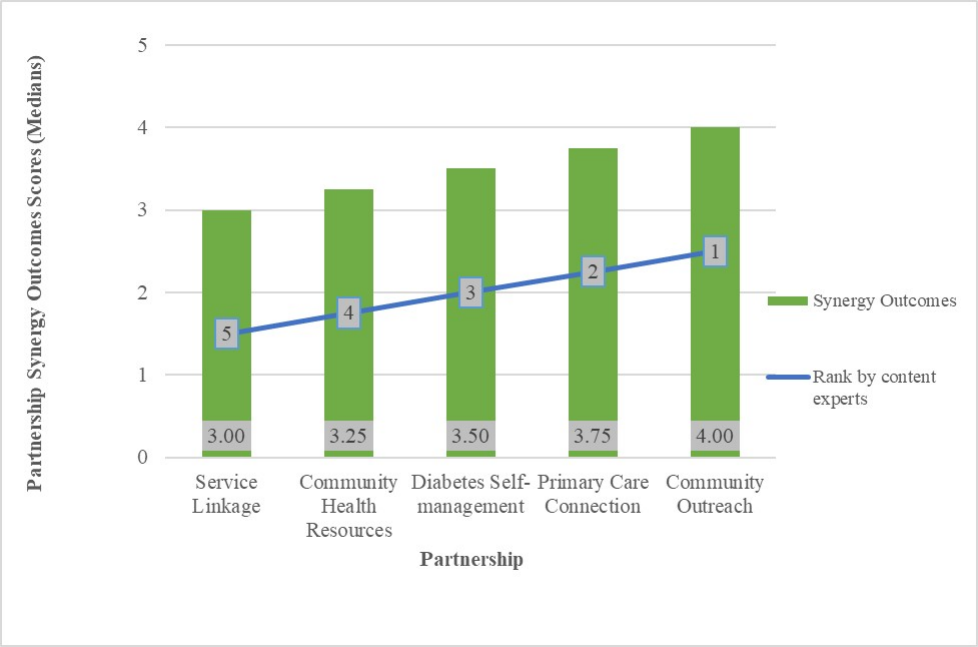
Partnership synergy outcomes (partnership-level median scores) and rankings of partnerships based on assessment by content reviewers (highest = 1 to lowest = 5) (*N = 5*).

We aggregated the scores at the partnership level for Partnership Synergy Outcomes, Partnership Synergy Processes, Partnership Synergy and the following dimensions of partnership functioning: decision-making, leadership, administration & management, non-financial resources, financial resources and resource utilization. We then ranked the five partnerships on the basis of their aggregate scores. Fig 1 displays the comparison of the ranking based on quantitative scores with the independent, qualitative rankings by the content reviewers. The quantitative and qualitative rankings were completely coherent for Partnership Synergy Outcomes, showing that partnership synergy was highest in the Community Outreach partnership and lowest in the Service Linkage partnership. Our one-way analysis of variance was suggestive of statistically significant differences between at least two of the medians of Partnership Synergy Processes (*P* = .09) and Partnership Synergy (*P* = .07). The differences in medians were statistically significant between the following partnerships: Primary Care Connection-Service Linkage, Community Outreach-Community Health Resources, Community Outreach-Diabetes Self-management and Community Outreach-Service Linkage (in decreasing order of magnitude of difference). To further explore the extent to which differences in aggregate scores were meaningful, we calculated and found low to moderate variation for partnership synergy in all partnerships, suggesting that the statistically significant Kruskal-Wallis test of difference between at least two partnerships was not spurious. There was some variation in the content reviewers’ ranking of partnership synergy processes and partnership synergy outcomes, that was not reflected in the quantitative scores (see supplementary material S3 Appendix), suggesting that the items may not have captured nuances in the construct.

**Fig 2:**
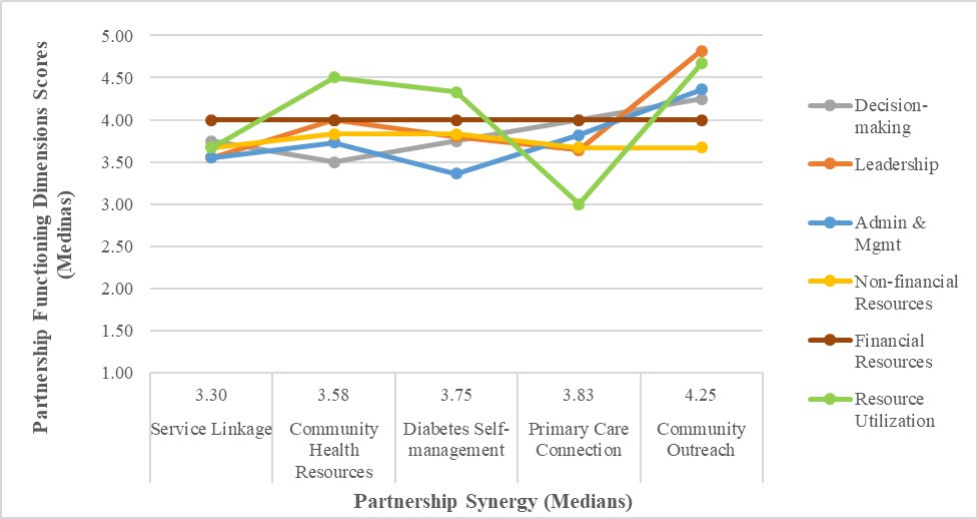
Partnership-level scores on dimensions of partnership functioning. Partnerships are ordered by increasing score on partnership synergy (and rank).

Fig 2 demonstrates that the partnership ranking was generally reflected in the scores of the partnership functioning dimensions, which was largely coherent with the results in Table 5 that show moderate to strong correlations between certain dimensions of partnership functioning and Partnership Synergy sub-scales. However, Fig 2 also displays a variation in ranking of the dimensions of partnership functioning, suggesting that partnership synergy is not simply another measure of partnership functioning.

Irrespective of the variations in ranking of the different dimensions of partnership functioning, the partnership-level scores for achieving the overall goal of developing a meaningful partnership were largely consistent with partnership-level partnership synergy scores. The majority of respondents in all partnerships reported that a meaningful partnership had been achieved leading to an uninformative median of 4 across all partnerships. However, as shown in Table 6, there was considerable variation within the partnerships that was reflected in the means and standard deviations. The metric that corresponds best to both partnership synergy scores and the qualitative ranking is the percentage endorsing that the partnership goal was achieved *very well or extremely well*.

**Table 6:** Partnership-level assessment of the extent to which the goal of developing a meaningful partnership has been achieved.

Partnership	Not well at all or not so well	Moderately well	Very well or extremely well	Mean (SD)
**Service Linkage (n = 9)**	2 (22.2%)	3 (33.3%)	4 (44.4%)	3.22 (1.20)
**Community Health Resources (n = 15)**	0	5 (33.3%)	10 (66.7%)	3.87 (0.74)
**Diabetes Self-management (n = 7)**	0	2 (28.6%)	5 (71.4%)	3.86 (0.69)
**Primary Care Connection (n = 10)**	1 (10%)	2 (20%)	7 (70%)	3.60 (0.70)
**Community Outreach (n = 7)**	0	1 (14.3%)	6 (85.7%)	4.14 (0.69)

## Discussion

This study demonstrated that an adapted version of the PSAT was capable of discriminating between different levels of dimensions of partnership functioning and partnership synergy even in a limited sample of ongoing partnerships working towards improving PHC. The measures are sufficiently reliable to have the capacity to discriminate between individuals’ perceptions of the partnerships, and between partnerships based on an aggregated score. The quantitative measures could not distinguish meaningfully between Partnership Synergy Processes and Partnership Synergy Outcomes, and we proposed a single measure of Partnership Synergy. Our new sub-scales on Communication and External Environment did not perform well metrically, but could be further developed. Our findings demonstrate that the PSAT can be a valuable evaluation tool for partnerships that are underway, in that it supports identification of the areas of strength and potential improvement to achieve the collaborative advantage over organizations working on their own, even before achievement of the planned ultimate outcomes can be assessed. The tool requires further refinement and possible elimination of redundancies in order to be administered at several points in the life of partnerships in PHC transformation.

We adapted the PSAT [22] on the basis of findings from a qualitative investigation that preceded this study. Some of the items were adapted based on qualitative codes, and new items were developed for Communication and External Environment. In the 2002 version of the PSAT, item content related to communication was found in the following scales: Synergy, Leadership, and Administration & Management [22]. While we retained these items in the Leadership and Administration & Management sub-scales, we also developed a separate sub-scale for the domain of communication, for our qualitative data demonstrated the importance of communication as a stand-alone dimension of partnership functioning [35]. Clear, targeted and timely communication via a variety of communication mechanisms was deemed by qualitative study participants to be one of the key drivers in ensuring transparency and enhancing stakeholder engagement. However, the three-item sub-scale that we designed did not perform well metrically based on assessment of item-convergent validity, item-discriminant validity, or internal consistency. This may be because we used a different response approach for each item: a descriptive range for the first question on ways stakeholders were informed about what is happening in the partnership; rating of usefulness of information received; and an assessment of adequacy of quantity of information. Despite various attempts to express the nominal descriptive item in ordinal categories of increasing magnitude, the exercise did not yield satisfactory results, and even after eliminating this poorly functioning item and retaining only two items the internal consistency remained low (0.49). However, the Spearman rank correlations between all three communication items and partnership synergy were statistically significant, different from zero and strong in the Primary Care Connection partnership (0.71) and the Community Health Resources partnership (0.51), upholding our qualitative conclusion that communication was an important dimension of partnership functioning. Future research should develop a more appropriate sub-scale to measure communication as an integral component of partnership functioning.

Similarly, for external environment, our qualitative findings demonstrated that external context had an important impact on the work of the partnerships in the case study [35]. The interventions in both partnerships under qualitative investigation unfolded within the context of significant changes in the respective health care systems. This contextual volatility necessitated ongoing adaptations to interventions to respond to evolving environmental opportunities and threats. However, our newly developed sub-scale on External Environment was not correlated with partnership synergy, nor any other dimension of partnership functioning. This finding is consistent with prior research. A study by Weiss, Anderson and Lasker (2002) revealed that community-related challenges, as measured by a three-item scale that the authors developed, did not have any relationship to synergy. The lack of correlation in our study might, however, have been due to the limitations of the scale used: the internal consistency of the scale that we developed was unacceptable (-0.74). This negative result might be due to the fact that it is easier to adapt to a smaller number of environmental changes, which would explain the reverse scoring of the two questions. Alternatively, the two items might not be measuring a single construct. The lack of correlation in our study may also be explained by the fact that external environment is more likely to influence how well the partnership achieves its specific objectives but less likely to have an impact on how the partnership achieves the goal of developing a meaningful partnership. In addition, external environment is highly variable and would require tailored adaptation strategies in each case.

Financial Resources was another sub-scale where we observed a low correlation with most other dimensions of partnership functioning and Partnership Synergy Outcomes. The three-item sub-scale assessed the degree to which the following resources were either present or absent: financial support, space, and equipment and goods. This finding was surprising given the results of prior research demonstrating the critical role of financial resources in partnership functioning [36]. In addition, the partnership synergy framework posits that money and other material resources are among the core determinants of synergy [3]. However, a later study by a team that included the authors behind the partnership synergy framework [19] looked at the relationship between partnership synergy and different dimensions of partnership functioning, and omitted financial resources as a dimension of interest. The authors stated that they had not expected money to be related to the ability of partners to achieve partnership synergy and that financial resources would more likely affect synergy indirectly, by enabling the presence of staff and resources to support the work of the partnership [19]. The weak correlation between Partnership Synergy Outcomes and Financial Resources in our study may be due to the limitations of our Partnership Synergy Outcomes sub-scale (Cronbach’s α < 0.70). The projects undertaken by partnerships in our sample were funded under the envelope of the larger IMPACT program of research. This funding covered the partnerships’ coordinating infrastructure/research support, including the partnership coordinator position in each site, as well as the evaluation of the interventions, but not intervention implementation. Partnership stakeholders, other than research coordinators, were not remunerated for the time spent on partnership activities. Each partnership was required to mobilize adequate local resources to respond to regional access needs and to maintain interventions beyond the life of the IMPACT research funding. Our qualitative study revealed the critical importance of the funded coordinating infrastructure, but that the efforts to secure funding for the interventions had variable success. However, our Financial Resources sub-scale did not make a distinction between different types of funding. This lack of granularity might have affected the uninformative median of 4 for Financial Resources across all five partnerships. In addition, most partnership stakeholders participated on a continuous voluntary basis which suggests that they were driven by considerations other than financial benefit. Future research should focus on further assessing the relationship between financial resources, partnership synergy and different partnership functioning dimensions.

### Synergy outcomes and processes

We assessed partnership synergy with two constructs with a limited number of items (two items from the Weiss *et al*. scale and five items from the Jones synergy scale). We were unable to detect a significant distinction between the two Partnership Synergy sub-scales in terms of how strongly each one of them correlated with other dimensions of partnership functioning. This finding is consistent with prior research that suggested that the Weiss *et al*. and Jones synergy scales could be used interchangeably [20]. On the other hand, our inability to distinguish between these sub-scales might be due to the limited number of items that we had retained. However, the qualitative ranking by independent content reviewers highlighted differences between the two constructs, suggesting the need for further development of separate partnership synergy processes and partnership synergy outcomes measures. Further assessment of the applicability of both scales is warranted. The scales could be administered longitudinally, at various stages in the partnership evolution, to determine if one is more applicable within the scope of partnership process improvements and the other–as part of outcome evaluation, and whether both scales yielding similar results only occurs at a certain point in partnership development.

At the partnership level, the partnership with the highest total partnership synergy score (Community Outreach) achieved on average the highest scores for partnership functioning on most dimensions. However, the results in the case of partnerships with lower scores on the Partnership Synergy and partnership functioning sub-scales were mixed. One partnership (Primary Care Connection) achieved a high synergy score despite a number of lower scores on the partnership functioning sub-scales, the most noticeable one of which was Resource Utilization. The sub-scale Resource Utilization was referred to as “Efficiency” in the 2002 version of the PSAT [22]. Efficiency was conceptualized as the degree to which a partnership optimized the involvement of its partners [3]. The three-item sub-scale assessed how well the partnership used partner’s time, other non-financial resources, and financial resources [22]. Our qualitative investigation revealed that the Primary Care Connection partnership (which scored lower than other partnerships on this sub-scale) experienced difficulty in meaningfully engaging community-based stakeholders from or representing the target population. In addition, the partnership witnessed reduced engagement of some stakeholders, as they reported that the opportunity cost of conducting activities on behalf of the partnership beyond the face-to-face meetings was too great. Moreover, the extent of environmental impact and adaptation in the case of the Primary Care Connection partnership was particularly profound. The partnership had to redesign the intervention several times to respond to evolving environmental opportunities and threats, and lost some key partners as their organizational positions had changed. These developments may have affected the perceived degree to which the use of financial resources and partners’ time were optimized. The relatively high synergy score for this partnership suggests that there might be certain dimensions that are still lacking from our adapted version of the PSAT and that partnership synergy is an important intermediate outcome worthy of more research. Taking into consideration the emphasis that the stakeholders in all five partnerships put on the quality of relationships, we recommend that future research focus on incorporating into a revised iteration of the PSAT an assessment of the quality of stakeholder relationships, including such aspects as trust, as a stand-alone scale or sub-scale.

### Practice implications

The PSAT is a generic tool that has demonstrated robustness in assessing the quality of partnership processes in multi-stakeholder partnerships in several areas, including public health [24]. The PSAT has also been identified as a valid tool for measuring group processes in interprofessional health and social service partnerships at the front-line service provider group level [24]. The original creators of the tool argue that it could have an even broader appeal, including partnerships outside of health care [23]. However, the tool was designed for internal evaluation and not for use by external evaluators [6].

The PSAT may need to be tailored to different partnership types and local conditions. For example, community-driven partnerships with fewer resources, a decentralized partnership structure and more distributed decision-making may need to adapt the Leadership and Administration & Management sub-scales. Alternatively, partnerships may prioritise different aspects for evaluation altogether. There is a variety of other partnership assessment tools that may be more or less suitable depending on the purpose of evaluation and the intended users of its results. For example, the Human Services Integration Measure [37] will be more useful in evaluating the structural components of partnerships, whereas the Team Climate Inventory [38]–for assessing the partnership’s climate for innovation.

The utility of the PSAT as a longitudinal measure of change has not yet been established [23]. We contend that once the PSAT tool is refined and validated, it can reasonably be administered at different times in the evolution of the partnership. The original creators of the PSAT caution that the tool is not intended to be administered at the partnership inception stage [23]. The partnerships should have reached a certain degree of maturity: have been in existence for at least six months, have taken steps to implement plans and be comprised of at least five stakeholders who have continually worked together [23]. For newly formed partnerships, other tools (e.g., the Partnership Analysis Tool developed by VicHealth [11] or the Verona Benchmark [12]) would be more appropriate, as an introduction to the basic concepts of partnership and a guide to structured partnership development.

Once the partnership composition is established, and the partners have had the chance to work together (including face-to-face meetings), the revised PSAT can be administered in order to assess how the partnership is evolving and identify any areas for improvement. It may take some partnerships longer than six months to create momentum and therefore the timing of initial administration needs to be determined based on the specific circumstances of each partnership. At the end of the project the tool would benefit from additional sub-scales on effectiveness and sustainability to assess the likelihood of sustainability and impact on the target populations. In longer projects, if time and resources permit, we recommend that the tool be also administered at the mid-project point, to quantify progress related to how the collaboration is working, and identify strengths and areas that would require more work. In multi-partnership projects the tool would have to be administered at relatively similar intervals to ensure comparability across sites.

Partnership self-assessment, including the administration of the tool, its analysis and sharing of results can be time-consuming, costly in terms of resources, and may contribute to participant burden [15,39]. These factors will affect decisions regarding the tool’s recurrent use. However, we are aware of at least one attempt to develop a shorter version of the PSAT [40], which may be more appropriate for a collaboration “health check” [10] at shorter intervals.

### Limitations

Our results should be interpreted in light of the study’s limitations. The first limitation concerns the generalizability of the study’s results. The sample of partnerships is small and the scope is narrow. All participants were sampled from a single international program of research with a targeted scope of implementing organizational interventions to improve the performance of accessibility to PHC, for a particular vulnerable population. Academic representatives constituted the largest single group of respondents in each partnership, which might have influenced our results. Despite the fact that most survey questions were fairly generic, they may have elicited different meaning for different participant groups [41]. For example, different stakeholders may have conceptualized success differently. In addition, it was revealed in the course of our qualitative investigation that academic stakeholders were more likely to have experienced a multi-stakeholder collaborative approach to problem solving and were more familiar with evaluation processes. This may have influenced their willingness to participate in this survey. Secondly, due to the cross-sectional study design, the causality and temporality of associations between the dimensions of partnership functioning and the level of partnership synergy achieved could not be inferred. Taking into consideration that we focused on evolving partnerships, we did not use a separate tool to measure partnership effectiveness. Therefore, we could not demonstrate definitively if partnership synergy was indeed a determinant of partnership effectiveness. In addition, this snapshot assessment might not have captured the holistic nature of partnerships as complex, constantly changing entities. Lastly, while being considered a strength in certain respects, developing the quantitative instrument in both English and French might have resulted in certain inconsistencies when it comes to translating specific terms.

We considered and employed a number of mitigation strategies to overcome some of these limitations. In order to address the issue of small sample size, we used recommended statistical procedures and supplemented our analyses with qualitative information by independent content reviewers. In terms of the scope of partnership activities, we supplemented our survey data with information generated throughout the qualitative phase that preceded this quantitative study. Despite the specificity of partnerships under investigation, there was enough heterogeneity among them, in terms of their contexts, their composition and how the partnerships evolved, to assess the utility of the tool in comparing partnership performance across various settings. In terms of possible translation limitations, we ensured the accuracy of terminology through cognitive testing. Enriching our analyses with qualitative data from the first phase of this larger study has also allowed us to counter some of the challenges involved in using standardized self-assessment tools to assess partnerships. With regards to the causal relationships between partnership functioning and synergy, the administration of a scale to assess the effectiveness of both processes and outcomes would be warranted as all partnerships have now completed their work. This would allow us to determine whether higher synergy actually leads to better sustainability of interventions and better outcomes for the target populations.

## Conclusions

We adapted an existing partnership self-assessment tool and administered it to a small sample of developing partnerships. Our findings demonstrate that the tool is suitable for assessing the achievement of partnership synergy and the performance of each partnership along specific indicators of partnership functioning. The tool allowed us to detect meaningful differences between partnerships in how they performed on various dimensions, and corresponded overall to independent qualitative evaluations. The instrument demonstrates good capacity to discriminate between partnerships, and could be applied to assess internal partnership performance on these key indicators across settings, in order to determine if their collaborative process is working well. Therefore, it may have broader applicability to other partnerships in PHC, and arguably beyond PHC. We also propose that additional constructs of quality of partner relationships, communication and external environment could be developed and validated as part of partnership assessment.

In conclusion, this study adds to the growing body of literature on assessing partnership synergy as a predictor of effectiveness of multi-stakeholder partnerships in health care, with a specific focus on PHC. In general, this work has contributed to the evolving body of knowledge on partnership synergy as a useful framework for studying collaborative ventures and identifying the main ingredients or requirements for synergistic partnerships. These requirements should be considered in order to determine if working in partnership should and can reasonably be pursued.

## Supporting information

S1 Appendix Comparison of questionnaires. (DOCX)

S2 Appendix Spearman’s rank individual correlations. (XLSX)

S3 Appendix Comparison of rankings of partnerships. (DOCX)

S1 Dataset Partnership questionnaire. (XLSX)
