# Supplementary material for: Measuring partnership synergy and functioning: Multi-stakeholder collaboration in primary health care
Source: PLoS One. 2021 May 28;16(5):e0252299. doi: 10.1371/journal.pone.0252299 (PMC8162647; doi:10.1371/journal.pone.0252299)
Supplement: S1 Appendix — (DOCX) [file pone.0252299.s001.docx]

**S1 Appendix. Comparison of Questionnaires.**

**Comparison of the Multi-Stakeholder Partnership Questionnaire (MSPQ) with**

**(a) the PSAT and (b) the Partner Questionnaire.**

| **MSP Study Questionnaire** | **PSAT / Partner Questionnaire** | **Changes Implemented** |
| --- | --- | --- |
| **Communication (new subscale in MSPQ)** | | |
| *Preamble: Please think about communication and information sharing in your partnership.* |  |  |
| **How do you learn about what is happening in the partnership?** *Check all that apply*  ☐ Updates during in-person meetings  ☐ Minutes of previous meetings  ☐ Electronic updates between meetings (e.g., newsletter)  ☐ Verbal unstructured information sharing by partners  ☐ External conferences and events  ☐ Other, please specify____ | **PSAT / Partner Questionnaire:** Not elicited | **New question, based on qualitative findings that highlighted the importance of communication to the functioning of partnerships** |
| **How useful is the information that you receive?**  ☐ Not very useful  ☐ A little useful  ☐ Moderately useful  ☐ Useful  ☐ Very useful  ☐ I do not recall receiving any information | Not elicited | **New question, based on qualitative findings** |
| **Do you receive enough information regarding partnership’s activities?**  ☐ No, but I do not need more information  ☐ No, and I would have liked more information  ☐ Yes, mostly I am adequately informed  ☐ Yes, I am always informed | Not elicited | **New question, based on qualitative findings** |
| **Decision-making** | | |
| *Preamble: Please think about how decisions are made and challenges are resolved in your partnership.* |  |  |
| **What are the top two ways in which decisions are made in the partnership?**  ☐ Consensus: discussion and then everyone agrees to support the decision  ☐ Majority rule: voting/show of hands  ☐ Executive: chair/leader decides  ☐ Expert: decision delegated to a small subgroup  ☐ Default: action/inaction forces a conclusion | **Partner Questionnaire:**  What is the **main** way decisions are made in the partnership? **PLEASE CIRCLE ONE ANSWER ONLY.**   \| Consensus (discussion and then everyone agrees) \| 5 \| \| --- \| --- \| \| Voting/show of hands \| 4 \| \| Official subcommittees decide \| 3 \| \| Chair/leader decides \| 2 \| \| Made by a few partners outside the partnership meetings \| 1 \| \| Don’t know \| 0 \| \| If other, please say what it is \| \| | **Response options modified, based on qualitative findings regarding how decisions were made during partnership face-to-face meetings** |
| **How comfortable are you with the way decisions are made in the partnership?**  ☐ Not very comfortable  ☐ A little comfortable  ☐ Moderately comfortable  ☐ Comfortable  ☐ Very comfortable | **PSAT:**  How comfortable are you with the way decisions are made in the partnership?  [ ] Extremely comfortable  [ ] Very comfortable  [ ] Somewhat comfortable  [ ] A little comfortable  [ ] Not at all comfortable | **Response options modified, based on cognitive testing, changed the valence of response options from negative (1) to positive (5),**  **in order to reduce the commonly observed positive bias in responding** |
| **How often do you feel that you are included in the decision-making process?**  ☐ None of the time  ☐ Almost none of the time  ☐ Some of the time  ☐ Most of the time  ☐ All of the time | **PSAT:**  How often do you feel that you have been left out of the decision making process?  [ ] All of the time  [ ] Most of the time  [ ] Some of the time  [ ] Almost none of the time  [ ] None of the time | **Modified question to use an appreciative stance, changed the valence of response options** |
| **How well does the partnership address problems?**  ☐ Not well at all  ☐ Not so well  ☐ Moderately well  ☐ Very well  ☐ Extremely well | **PSAT and Partner Questionnaire:** Problem-solving questions were part of the two synergy scales. | **New question, based on qualitative findings, moved to the decision-making section in order to reflect the critical thinking process involved in problem solving and decision-making** |
| **Leadership** | | |
| *Preamble: Please think about all the people who provide either formal or informal leadership in your partnership.* | **PSAT:**  *Preamble: Please think about all of the people who provide either formal or informal leadership in this partnership.* |  |
| **How would you rate your partnership’s leadership in each of the following areas:**  **-** Taking responsibility for the partnership  - Communicating the vision of the partnership  - Working to develop a common language within the partnership  - Building on the perspectives,  resources and skills of partners  **-** Helping the partnership to look at things differently  - Fostering respect, trust, inclusiveness and openness in the partnership  - Creating an environment where differences of opinion can be voiced  - Inspiring or motivating people involved in the partnership  - Empowering people involved in the partnership  **-** Resolving conflict among partners  - Recruiting diverse people and organizations into the partnership  **All response options: Poor, Fair, Good, Very Good, Excellent** | Please rate the total effectiveness of your partnership’s leadership in each of the following areas:  a. Taking responsibility for the partnership  b. Inspiring or motivating people involved in the partnership  c. Empowering people involved in the partnership  d. Communicating the vision of the partnership  e. Working to develop a common language within the partnership  f. Fostering respect, trust, inclusiveness, and openness in the partnership  g. Creating an environment where differences of opinion can be voiced  h. Resolving conflict among partners  i. Combining the perspectives, resources, and skills of partners  j. Helping the partnership be creative and look at things differently  k. Recruiting diverse people and organizations into the partnership  **All response options: Excellent, Very good, Good, Fair, Poor, Don’t know** | **Modified the introductory question eliminating reference to “total effectiveness” due to various conceptualizations of effectiveness; modified the order of component questions; changed the valence of response options; removed “don’t know” to force a response; changed into a matrix to reduce the amount of space used** |
| **Administration/Management** | | |
| *Preamble: Within the framework of IMPACT the administrative and management activities in your partnership have mostly been undertaken by the research teams.* | **PSAT:**  *Preamble: We would like you to think about the administrative and management activities in your partnership.* |  |
| **How would you rate the administration/management’s performance in each of the following areas:**  **-** Coordinating communication among partners  - Coordinating communication with people and organizations outside the partnership  - Informing partners on how their input is being integrated  - Organizing the overall work of the partnership  - Performing secretarial duties  - Preparing materials that inform partners and help them make timely decisions  - Providing orientation to new partners as they join the partnership  - Minimizing barriers to participation in meetings and activities (e.g., meetings at convenient places and times, providing transportation, etc.)  - Facilitating meetings  - Accurately reflecting discussions and decisions in the minutes of meetings or other documentation  - Evaluating the progress and impact of the partnership  **All response options: Poor, Fair, Good, Very Good, Excellent** | Please rate the effectiveness of your partnership in carrying out each of the following activities:  a. Coordinating communication among partners  b. Coordinating communication with people and organizations outside the partnership  c. Organizing partnership activities, including meetings and projects  d. Applying for and managing grants and funds  e. Preparing materials that inform partners and help them make timely decisions  f. Performing secretarial duties  g. Providing orientation to new partners as they join the partnership  h. Evaluating the progress and impact of the partnership  i. Minimizing the barriers to participation in the partnership’s meetings and activities (e.g., by holding them at convenient places and times, and by providing transportation and childcare)  **All response options: Excellent, Very good, Good, Fair, Poor, Don’t know** | **Modified the introductory question eliminating reference to “effectiveness” due to its various conceptualizations; modified the order of component questions; changed the valence of response options; removed “don’t know” to force a response; changed into a matrix to reduce the amount of space used; removed “Applying for and managing grants and funds” as it was not relevant to the partnerships being studied; added “Accurately reflecting discussions and decisions in the minutes of meetings or other documentation” from the Partner Questionnaire; added**  **“Informing partners on how their input is being integrated”**  **as qualitative data highlighted the importance of feedback loops** |
| **Non-financial resources** | | |
| *Preamble: A partnership needs non-financial resources in order to work effectively and achieve its goals.* | **PSAT:**  *Preamble: A partnership needs non-financial resources in order to work effectively and achieve its goals.* |  |
| **To what extent does your partnership have the skills and expertise to work well (e.g., leadership, administration, evaluation, law, public policy, cultural competency, training, etc.)?**  ☐ None of what it needs  ☐ Little of what it needs  ☐ Some of what it needs  ☐ Most of what it needs  ☐ All of what it needs | For each of the following types of resources, to what extent does your partnership have what it needs to work effectively?  a. Skills and expertise (e.g., leadership, administration, evaluation, law, public policy, cultural competency, training, community organizing):  [ ] All of what it needs  [ ] Most of what it needs  [ ] Some of what it needs  [ ] Almost none of what it needs  [ ] None of what it needs  [ ] Don’t know | **Re-wrote as separate questions; modified the response options; removed “don’t know” to force a response** |
| **To what extent does your partnership have the data and information to work well (e.g., statistical data, information about community perceptions, values, resources and politics)?**  ☐ None of what it needs  ☐ Little of what it needs  ☐ Some of what it needs  ☐ Most of what it needs  ☐ All of what it needs | b. Data and information (e.g., statistical data, information about community perceptions, values, resources and politics)?  [ ] All of what it needs  [ ] Most of what it needs  [ ] Some of what it needs  [ ] Almost none of what it needs  [ ] None of what it needs  [ ] Don’t know |  |
| **To what extent does your partnership have the connections to target populations to work well?**  ☐ None of what it needs  ☐ Little of what it needs  ☐ Some of what it needs  ☐ Most of what it needs  ☐ All of what it needs | c. Connections to target populations  [ ] All of what it needs  [ ] Most of what it needs  [ ] Some of what it needs  [ ] Almost none of what it needs  [ ] None of what it needs  [ ] Don’t know |  |
| **To what extent does your partnership have the connections to political decision-makers, government agencies, other organizations/groups to work well?**  ☐ None of what it needs  ☐ Little of what it needs  ☐ Some of what it needs  ☐ Most of what it needs  ☐ All of what it needs | d. Connections to political decision-makers, government agencies, other organizations/groups  [ ] All of what it needs  [ ] Most of what it needs  [ ] Some of what it needs  [ ] Almost none of what it needs  [ ] None of what it needs  [ ] Don’t know |  |
| **To what extent does your partnership have legitimacy and credibility to work well?**  ☐ None of what it needs  ☐ Little of what it needs  ☐ Some of what it needs  ☐ Most of what it needs  ☐ All of what it needs | e. Legitimacy and credibility  [ ] All of what it needs  [ ] Most of what it needs  [ ] Some of what it needs  [ ] Almost none of what it needs  [ ] None of what it needs  [ ] Don’t know |  |
| **To what extent does your partnership have the convening power (influence and ability to bring people together for meetings and activities) to work well?**  ☐ None of what it needs  ☐ Little of what it needs  ☐ Some of what it needs  ☐ Most of what it needs  ☐ All of what it needs | f. Influence and ability to bring people together for meetings and activities  [ ] All of what it needs  [ ] Most of what it needs  [ ] Some of what it needs  [ ] Almost none of what it needs  [ ] None of what it needs  [ ] Don’t know |  |
| **Financial and other resources** | | |
| *Preamble: A partnership also needs financial and other capital resources in order to work effectively and achieve its goals.* | **PSAT:**  *Preamble: A partnership also needs financial and other capital resources in order to work effectively and achieve its goals.* |  |
| **To what extent does your partnership have financial support that it needs to work well?**  ☐ None of what it needs  ☐ Little of what it needs  ☐ Some of what it needs  ☐ Most of what it needs  ☐ All of what it needs | For each of the following types of resources, to what extent does your partnership have what it needs to work effectively?  a. Money  [ ] All of what it needs  [ ] Most of what it needs  [ ] Some of what it needs  [ ] Almost none of what it needs  [ ] None of what it needs  [ ] Don’t know | **Re-wrote as separate questions; modified the response options; removed “don’t know” to force a response** |
| **To what extent does your partnership have the space that it needs to work well?**  ☐ None of what it needs  ☐ Little of what it needs  ☐ Some of what it needs  ☐ Most of what it needs  ☐ All of what it needs | b. Space  [ ] All of what it needs  [ ] Most of what it needs  [ ] Some of what it needs  [ ] Almost none of what it needs  [ ] None of what it needs  [ ] Don’t know |  |
| **To what extent does your partnership have the equipment and goods that it needs to work well?**  ☐ None of what it needs  ☐ Little of what it needs  ☐ Some of what it needs  ☐ Most of what it needs  ☐ All of what it needs | c. Equipment and goods  [ ] All of what it needs  [ ] Most of what it needs  [ ] Some of what it needs  [ ] Almost none of what it needs  [ ] None of what it needs  [ ] Don’t know |  |
| **Resource utilization (Efficiency in PSAT and Partner Questionnaire)** | | |
| *Preamble: Please think about the degree to which various partnership resources are optimized.* |  |  |
| **Please choose the statement that best describes how well your partnership uses its non-financial resources (e.g., skills, expertise, information, data, connections, influence, space, equipment, goods, etc.).**  ☐ The partnership makes poor use of its non-financial resources  ☐ The partnership makes fair use of its non-financial resources  ☐ The partnership makes good use of its non-financial resources  ☐ The partnership makes very good use of its non-financial resources  ☐ The partnership makes excellent use of its non-financial resources  ☐ I do not know how the partnership uses its non-financial resources | **PSAT:**  Please choose the statement that best describes how well your partnership uses the partners’ in-kind resources (e.g., skills, expertise, information, data, connect ions, influence, space, equipment, goods).  [ ] The partnership makes excellent use of partners’ in-kind resources.  [ ] The partnership makes very good use of partners’ in-kind resources.  [ ] The partnership makes good use of partners’ in-kind resources.  [ ] The partnership makes fair use of partners’ in-kind resources.  [ ] The partnership makes poor use of partners’ in-kind resources. | **Changed from “in-kind resources” to “non-financial resources” as “in-kind” would not be understood by all partners; changed the valence of response options; added “don’t know” as a response option for all questions, as some partners in the partnerships under investigations were not involved in decisions regarding resources; added “not applicable” as a response option in question regarding financial resources as some partnerships did not have financial resources** |
| **Please choose the statement that best describes how well your partnership uses the partners’ time.**  ☐ The partnership makes poor use of partners’ time  ☐ The partnership makes fair use of partners’ time  ☐ The partnership makes good use of partners’ time  ☐ The partnership makes very good use of partners’ time  ☐ The partnership makes excellent use of partners’ time  ☐ I do not know how the partnership uses partners’ time | Please choose the statement that best described how well your partnership uses the partners’ time.  [ ] The partnership makes excellent use of partners’ time.  [ ] The partnership makes very good use of partners’ time.  [ ] The partnership makes good use of partners’ time.  [ ] The partnership makes fair use of partners’ time.  [ ] The partnership makes poor use of partners’ time. |  |
| **Please choose the statement that best describes how well your partnership uses its financial resources, including those of any partner.**  ☐ The partnership makes poor use of its financial resources  ☐ The partnership makes fair use of its financial resources  ☐ The partnership makes good use of its financial resources  ☐ The partnership makes very good use of its financial resources  ☐ The partnership makes excellent use of its financial resources  ☐ I do not know how the partnership uses its financial resources  ☐ Not applicable, there are no financial resources | Please choose the statement that best describes how well your partnership uses the partners’ financial resources.  [ ] The partnership makes excellent use of partners’ financial resources.  [ ] The partnership makes very good use of partners’ financial resources.  [ ] The partnership makes good use of partners’ financial resources.  [ ] The partnership makes fair use of partners’ financial resources.  [ ] The partnership makes poor use of partners’ financial resources. |  |
| **External environment (new subscale in MSPQ)** | | |
| *Preamble: Please think about all the external influences that your partnership has experienced (e.g., changes in the funding landscape, policy changes) that have had an impact on the partnership or achievement of its goals.* |  |  |
| **To what extent has the partnership been affected by external factors, beyond the control of the partnership?**  ☐ Not at all  ☐ A little  ☐ Moderately  ☐ A lot  ☐ A great deal | **PSAT / Partner Questionnaire:** No separate external environment scale | **New question, based on qualitative findings that highlighted the important impact of the external environment on the work of the partnerships** |
| **How well has the partnership adapted to these external influences?**  ☐ Not well at all  ☐ Not so well  ☐ Moderately well  ☐ Very well  ☐ Extremely well |  | **New question, based on qualitative findings** |
| **Partnership’s Value (Synergy in PSAT)** | | |
| *Preamble: Please think about the people and organizations that are participants in your partnership.* | **PSAT:**  *Preamble: Please think about the people and organizations that are participants in your partnership.* |  |
| **By working together, how well are these partners able to identify new and creative ways to solve problems?**  ☐ Not well at all  ☐ Not so well  ☐ Moderately well  ☐ Very well  ☐ Extremely well | By working together, how well are these partners able to identify new and creative ways to solve problems?  [ ] Extremely well  [ ] Very well  [ ] Somewhat well  [ ] Not so well  [ ] Not well at all | **Outcome measure. Item from the Weiss synergy scale: changed valence of response options, changed “somewhat” to “moderately”** |
| **By working together, how well are these partners able to include the views and priorities of the people affected by the partnership’s work?**  ☐ Not well at all  ☐ Not so well  ☐ Moderately well  ☐ Very well  ☐ Extremely well | By working together, how well are these partners able to include the views and priorities of the people affected by the partnership’s work?  [ ] Extremely well  [ ] Very well  [ ] Somewhat well  [ ] Not so well  [ ] Not well at all | **Outcome measure. Item from the Weiss synergy scale: changed valence of response options, changed “somewhat” to “moderately”** |
| *Preamble: Now, thinking about how often things happen.* |  |  |
| **When working together, how often do you feel that the partnership is making good progress towards its goals?**  ☐ Never  ☐ Rarely  ☐ Sometimes  ☐ Often  ☐ Always | **Partner Questionnaire:**  When **working together**, how often does your partnership experience the following?  The partnership is making ongoing progress towards its goals  - Always  - Often  - Sometimes  - Rarely  - Never  - Don’t know | **Process measure. Item from the Jones synergy scale: modified question, changed from matrix question into a separate question, changed response option valence, removed “don’t know” response option** |
| **When working together, how often do you feel that added value is achieved as a result of working in this partnership?**  ☐ Never  ☐ Rarely  ☐ Sometimes  ☐ Often  ☐ Always | Extra outcomes are achieved as a result of working in partnership  - Always  - Often  - Sometimes  - Rarely  - Never  - Don’t know | **Process measure. Item from the Jones synergy scale: modified question, changed from matrix question into a separate question, changed response option valence, removed “don’t know” response option** |
| **When working together, how often do you feel that the skills and unique perspectives of the partners complement each other?**  ☐ Never  ☐ Rarely  ☐ Sometimes  ☐ Often  ☐ Always | The skills and unique perspectives of the partners complement each other  - Always  - Often  - Sometimes  - Rarely  - Never  - Don’t know | **Process measure. Item from the Jones synergy scale: changed from matrix question into a separate question, changed response option valence, removed “don’t know” response option** |
| **When working together, how often do you feel that all partners are benefiting from the activities of the partnership?**  ☐ Never  ☐ Rarely  ☐ Sometimes  ☐ Often  ☐ Always | All partners are benefiting from the activities of the partnership  - Always  - Often  - Sometimes  - Rarely  - Never  - Don’t know | **Process measure. Item from the Jones synergy scale: changed from matrix question into a separate question, changed response option valence, removed “don’t know” response option** |
| **When working together, how often does the partnership experience feelings of energy, excitement and passion?**  ☐ Never  ☐ Rarely  ☐ Sometimes  ☐ Often  ☐ Always | Feelings of energy, excitement and passion  - Always  - Often  - Sometimes  - Rarely  - Never  - Don’t know | **Process measure. Item from the Jones synergy scale: changed from matrix question into a separate question, changed response option valence, removed “don’t know” response option** |
| **Benefits** | | |
| *Preamble: Please think about the benefits of participating in this partnership for the organization that you are representing.* |  |  |
| **For each of the following benefits, please indicate whether the organization that you are representing has benefitted from:**  **-** Enhanced ability to address an important issue  - Development of new skills  - Heightened public profile of the organization  - Increased understanding of partners of my organization’s expertise and services  - Acquisition of useful knowledge about services, programs or people represented in the partnership  - Enhanced ability to affect public policy  - Development of valuable relationships  - Enhanced ability to meet the needs of my organization’s constituency or clients  - Ability to have a greater impact than we could have on our own  - Ability to make a contribution to the community  - Acquisition of additional financial support  **All response options: No, not at all; No, not really; Yes, a little; Yes, definitely** | **PSAT:**  For each of the following benefits, please indicate whether you have or have not received the benefit as a result of participating in the partnership:  a. Enhanced ability to address an important issue  [ ] Yes  [ ] No  b. Development of new skills  [ ] Yes  [ ] No  c. Heightened public profile  [ ] Yes  [ ] No  d. Increased utilization of my expertise or services  [ ] Yes  [ ] No  e. Acquisition of useful knowledge about services, programs, or people in the community  [ ] Yes  [ ] No  f. Enhanced ability to affect public policy  [ ] Yes  [ ] No  g. Development of valuable relationships  [ ] Yes  [ ] Np  h. Enhanced ability to meet the needs of my constituency or clients  [ ] Yes  [ ] No  i. Ability to have a greater impact that I could have on my own  [ ] Yes  [ ] No  j. Ability to make a contribution to the community  [ ] Yes  [ ] No  k. Acquisition of additional financial support  [ ] Yes  [ ] No | **Modified the introductory question changing emphasis from “you” to “organization”; changed the order of component questions; changed from stand alone questions into a matrix to reduce the amount of space used; changed the number of response option from Yes/No to No, not at all; No, not really; Yes, a little; Yes, definitely, in order to capture a more nuanced picture. Modified wording in questions c, d, e, h and i.** |
| **Drawbacks of participation** | | |
| *Preamble: Please think about the drawbacks of participating in this partnership for the organization that you are representing.* |  |  |
| **For each of the following drawbacks, please indicate whether the organization that you are representing has experienced the following:**  - Diversion of time and resources away from other priorities or obligations  - Insufficient influence in partnership activities  - Negative publicity due to association with other partners or the partnership  - Insufficient credit given to my organization for contributing to the accomplishments of the partnership  - Conflict between the demands of this partnership and other projects within my organization  **All response options: No, not at all; No, not really; Yes, a little; Yes, definitely** | **PSAT:**  For each of the following drawback, please indicate whether or not you have or have not experienced the drawback as a result of participating in this partnership.  a. Diversion of time and resources away from other priorities or obligations  [ ] Yes  [ ] No  b. Insufficient influence in partnership activities  [ ] Yes  [ ] No  c. Viewed negatively due to association with other partners or the partnership  [ ] Yes  [ ] No  d. Frustration or aggravation  [ ] Yes  [ ] No  e. Insufficient credit given to me for contributing to the accomplishments of the partnership  [ ] Yes  [ ] No  f. Conflict between my job and the partnership’s work  [ ] Yes  [ ] No | **Modified the introductory question changing emphasis from “you” to “organization”; changed from stand alone questions into a matrix to reduce the amount of space used; changed the number of response option from Yes/No to No, not at all; No, not really; Yes, a little; Yes, definitely, in order to capture a more nuanced picture. Modified wording in questions c, e, f; removed d.** |
| **Comparing benefits and drawbacks** | | |
| **On the whole, how do the benefits of participating in this partnership compare to the drawbacks?**  ☐ Benefits greatly exceed the drawbacks  ☐ Benefits exceed the drawbacks  ☐ Benefits and drawbacks are about equal  ☐ Drawbacks exceed the benefits  ☐ Drawbacks greatly exceed the benefits | **PSAT:**  So far, how have the benefits of participating in this partnership compared to the drawbacks?  [ ] Benefits greatly exceed the drawbacks  [ ] Benefits exceed the drawbacks  [ ] Benefits and drawbacks are about equal  [ ] Drawbacks exceed the benefits  [ ] Drawbacks greatly exceed the benefits | **Slightly modified the question, emphasizing the experience as a whole** |
| **Other comments** | | |
| **Overall, how well has the goal of developing a meaningful partnership been achieved?**  ☐ Not well at all  ☐ Not so well  ☐ Moderately well  ☐ Very well  ☐ Extremely well |  | **New question, to capture the aggregate assessment** |
