# Supplementary material for: Measuring partnership synergy and functioning: Multi-stakeholder collaboration in primary health care
Source: PLoS One. 2021 May 28;16(5):e0252299. doi: 10.1371/journal.pone.0252299 (PMC8162647; doi:10.1371/journal.pone.0252299)
Supplement: S3 Appendix — (DOCX) [file pone.0252299.s003.docx]

**S4 Appendix. Comparison of Quantitative and Qualitative Rankings of Partnerships -**

**Partnership Synergy Processes.**

**Table 1. Synergy processes (partnership-level mean scores (SD) and median scores (IQR)), and rankings of partnerships based on partnership synergy processes scores and qualitative assessment by content reviewers (*N=5*)**

| **Partnership** | **Partnership Synergy Processes (means and SD)** | **Partnership Synergy Processes (medians and IQR)** | **Rank by mean^a^ score,**  **Highest (1), lowest (5)** | **Rank by content reviewers,**  **Highest (1), lowest (5)** |
| --- | --- | --- | --- | --- |
| **Service Linkage** | 3.31 (0.75) | 3.60 (1.30) | 5 | 4 |
| **Community Health Resources** | 3.76 (0.53) | 3.60 (0.95) | 3 | 3 |
| **Diabetes Self-management** | 3.54 (0.61) | 3.80 (1.00) | 4 | 5 |
| **Primary Care Connection** | 3.78 (0.75) | 4.00 (1.00) | 2 | 2 |
| **Community Outreach** | 4.20 (0.37) | 4.20 (0.60) | 1 | 1 |
| ^a^ Mean values were used where there was insufficient differentiation between partnerships based on medians. | | | | |

**Figure 1: Partnership synergy processes (partnership-level mean scores) and rankings of partnerships based on qualitative assessment by content reviewers (highest = 1 to lowest = 5) (*N=5*)**
